# Comparative Study of Cytokine Measurements in Blood Plasma and Serum, and Saliva of Juvenile Pigs During Experimentally Induced Acute Inflammation

**DOI:** 10.3390/vetsci13010068

**Published:** 2026-01-09

**Authors:** Pernille Aagaard Madsen, Kevin Jerez-Bogotá, Darya Vodolazska, Charlotte Lauridsen

**Affiliations:** Department of Animal and Veterinary Sciences, Research Centre Foulum, AU Viborg, Aarhus University, Blichers Allé 20, 8830 Tjele, Denmark

**Keywords:** LPS infusion, systemic inflammation, cytokines, serum, saliva

## Abstract

This study aimed to assess cytokine levels in blood plasma and serum, and saliva of juvenile pigs in response to acute systemic inflammation. The objectives were to: (1) validate an analytical method for quantifying cytokines in serum; (2) assess the reliability of serum compared to plasma for cytokine quantification; and (3) explore the potential of saliva as a non-invasive alternative for cytokine measurement. Changes in 13 cytokines (IFN-γ, TNF-α, IL-1α, IL-1β, IL-1ra, IL-2, IL-4, IL-6, IL-8, IL-10, IL-12, IL-18 and GM-CSF) were analyzed in serum and saliva samples collected over a 72 h period following lipopolysaccharide (LPS) infusion to induce an acute inflammatory response in 10 juvenile pigs (~28 kg BW). EDTA plasma was collected over the same time period, and a subset of four cytokines (IL-1β, IL-6, IL-10 and IFN-γ) was analyzed to assess correlations with serum concentrations. A strong positive correlation was observed between serum and EDTA plasma levels of IL-1β, IL-6, IL-10 and IFN-γ (r = 0.91–1.00, *p* < 0.001), indicating that both serum and EDTA plasma can be used to obtain reliable measurements of cytokine concentrations in blood of juvenile pigs. Among the 13 analyzed cytokines in serum, TNF-α and IL-6 appeared as the most reliable cytokines during acute inflammation, peaking at 1 h and between 2 and 3 h post LPS infusion, respectively. In general, saliva did not correlate with serum for most cytokines, suggesting limited application of such a non-invasive matrix for systemic cytokine monitoring. However, IL-1α was detected at higher concentrations in saliva than in serum, suggesting that saliva may be useful for monitoring specific cytokines under certain inflammatory conditions. Further research is needed to clarify the origin and physiological role of salivary cytokines following LPS stimulation. Serum and plasma were suitable for cytokine analysis; however, serum may offer practical advantages by facilitating blood sample handling. Saliva may be useful for monitoring specific cytokines under certain inflammatory conditions.

## Simple Summary

Changes in concentrations of 13 cytokines were measured in serum and saliva samples collected over a 72 h period following lipopolysaccharide (LPS) infusion to induce an acute inflammatory response. A strong positive correlation was observed between serum and EDTA plasma concentrations, indicating that either serum or EDTA plasma can be used to obtain reliable measurements of cytokine levels in blood of juvenile pigs. In general, saliva did not correlate with serum for most cytokines, suggesting limited application of such a non-invasive matrix for systemic cytokine monitoring. However, IL-1α was detected at higher concentrations in saliva than in serum, suggesting that saliva may be useful for monitoring specific cytokines under certain inflammatory conditions. In conclusion, serum and plasma were suitable for cytokine analysis, while saliva may be useful for monitoring specific cytokines under certain inflammatory conditions.

## 1. Introduction

Cytokines are signaling proteins released during inflammatory responses that mediate communication between immune cells [1]. Overproduction of pro-inflammatory cytokines can cause tissue damage, including intestinal mucosal injury, and negatively impact growth performance [2,3]. For instance, pigs infected with *E. coli* showed reduced feed intake, weight gain and gain to feed ratio compared to non-infected pigs [4].

Cytokine concentrations in blood are commonly measured in veterinary research as biomarkers of disease status or to evaluate the efficacy of interventions targeting inflammation [5]. In porcine *E. coli* inoculation models [6], serum is preferred over plasma due to its routine use in clinical practice. Previous studies have investigated cytokine responses in pigs during acute inflammation with lipopolysaccharide (LPS) challenge being a widely established model to induce systemic activation [7].

LPS, a structural component of the outer membrane of Gram-negative bacteria, is a potent activator of the immune system [8]. Biological and physiological effects of LPS include activation of the immune system, synthesis of cytokines, increase in body temperature, reduced feed intake and alterations in acute phase protein concentration [9]. LPS stimulates macrophages to synthesize and secrete pro-inflammatory cytokines [10]. Hence, the LPS challenge is widely considered an effective model for investigating acute inflammatory responses in various animal models, including pigs [7]. LPS-based models in rodents have been developed to reproduce key features of human sepsis and offer several advantages, including technical simplicity and high reproducibility, particularly with respect to the induced inflammatory response [11]. Following LPS administration, circulating concentrations of pro-inflammatory cytokines increase markedly and can be readily measured in blood samples [12]. Among experimental models of systemic inflammation, the LPS model is widely considered the most suitable for evaluating the effects of new treatments on acute inflammatory responses [11].

Measuring cytokines in pig models, for example, to evaluate dietary interventions aimed at preventing or treating bacterial infections, can be challenging due to cytokine concentrations in blood may be present below the detection limit [13]. Several factors contribute to this issue, including insufficient cytokine responses in the measured matrix or incorrect timing of sample collection relative to the cytokine response. For example, in porcine models with *E. coli* inoculation, where the infection is present in the gut, cytokine levels in the blood may remain low if the infection is not systemically extended [14]. Additionally, cytokines analyzed in blood samples from pigs challenged with *E. coli* LPS peak at different time points [15], which must be considered when collecting samples for analysis. While blood plasma or serum are the most commonly used matrices to monitor inflammatory responses [15,16,17], little attention has been devoted to the application of saliva [18,19], which has the benefit of being a non-invasive sampling method.

The aim of this study was to apply a porcine LPS-induced inflammation model to: (1) validate an analytical method for the quantification of serum cytokines: Interferon gamma (IFN-γ), Tumor necrosis factor alpha (TNF-α), Interleukin-1 alpha (IL-1α), Interleukin-1 beta (IL-1β), Interleukin-1 receptor antagonist (IL-1ra), Interleukin-2 (IL-2), Interleukin-4 (IL-4), Interleukin-6 (IL-6), Interleukin-8 (IL-8), Interleukin-10 (IL-10), Interleukin-12 (IL-12), Interleukin-18 (IL-18) and Granulocyte-macrophage colony-stimulating factor (GM-CSF); (2) compare cytokine concentrations (IL-1β, IL-6, IL-10 and IFN-γ) between serum and EDTA plasma; and (3) evaluate the reliability of saliva as an alternative biological matrix to serum and EDTA plasma for cytokine quantification. It was hypothesized that cytokine levels in serum and EDTA plasma would be strongly positively correlated. It was further hypothesized that cytokine levels in saliva would show positive correlations with those in serum, indicating its potential as a reliable, non-invasive alternative matrix for cytokine assessment.

## 2. Materials and Methods

This study was based on an LPS challenge experiment previously described in Madsen et al. [20]. All procedures involving animals were reviewed and approved by the Danish Animal Experiments Inspectorate (License no. 2023-15-0201-01490) and the study complied with the ARRIVE guidelines [21].

### 2.1. Animals and Experimental Procedure

Ten clinically healthy, ~12-week-old female pigs [(Landrace × Yorkshire) × Duroc] with an average BW of 27.9 ± 0.77 kg were included. Female pigs were selected exclusively to control for possible confounding effects of sex on inflammatory responses. Animals were individually housed in pens without bedding to avoid contamination, provided with enrichment, and offered a commercial weaner diet (SmåGris Plus BF, DLG Tjele, Denmark) morning and evening. Water was available ad libitum. Health status, feed intake, and BW were monitored throughout the study.

A central venous catheter (2.2 mm FlexTip^®^, Teleflex, Reading, PA, USA) was placed in the jugular vein under general anesthesia (1 mL/15 kg Zoletil mix: Zoletil (Virbach Danmark A/S, Kolding, Denmark), butorphanol (Zoetis, Copenhagen, Denmark), ketamine (MSD Animal Health A/S, Copenhagen, Denmark), and xylazine (Elanco Denmark ApS, Ballerup, Denmark). Catheterization was performed using the Seldinger technique, and pigs were allowed a 24 h recovery period before the experimental procedures.

Pigs were randomly assigned to receive an intravenous infusion of *E. coli* LPS (O111:B4, Sigma-Aldrich, Darmstadt, Germany) at either 0.75 µg/kg BW (LOW) or 1.50 µg/kg BW (MODERATE), diluted in sterile 0.9% NaCl to 10 µg/mL. Following infusion, catheters were flushed with 10 mL sterile saline.

### 2.2. Sample Collection

**Figure 1:**
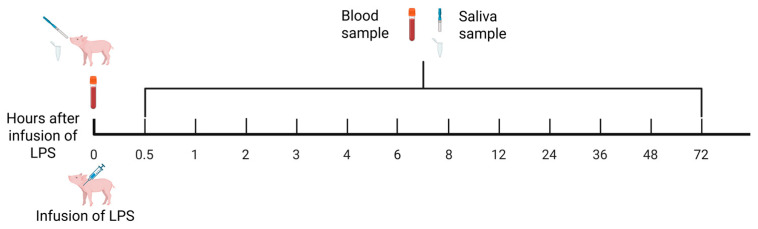
Overview of collection of blood and saliva samples during the experiment.

Blood and saliva samples were collected at 0 (pre-infusion), 0.5, 1, 2, 3, 4, 6, 8, 12, 24, 36, 48 and 72 h post-infusion (Figure 1).

Saliva samples were collected prior to the first blood sampling (time 0) and subsequently after each blood collection, as illustrated in Figure 1. Briefly, blood samples were collected in serum and EDTA plasma tubes at one baseline time point before LPS infusion and at 12 additional time points following the LPS infusion. After inverting the tubes 10 times, they were incubated on a roller mixer for 5 min. Plasma tubes were centrifuged immediately at 1500× *g* and 4 °C for 10 min. Serum tubes were allowed to stand for 30 min before centrifugation at 2000× *g* and 25 °C for 10 min. Following centrifugation, blood samples were transferred to microtubes and stored at −20 °C. Saliva samples were collected using the Salivette saliva collection kit (SARSTEDT, Skanderborg, Denmark) by using a long hemostat with a cotton and letting the pig chew on the cotton for approximately 30 s (until soaked). The cotton was placed in the Salivette tube and then centrifuged at 1000× *g* and 4 °C for 2 min. After centrifugation, saliva was pipetted into microtubes and immediately placed on ice. An inhibitor, a mix of BHT (Sigma-Aldrich, Søborg, Denmark) and EDTA (Sigma-Aldrich, Søborg, Denmark) to a final concentration of 0.2 mg/mL, was added to the saliva sampling tube to prevent degradation of analytes. All samples were stored at −70 °C until analyses.

### 2.3. Laboratory Analyses

Cytokine concentrations were measured in serum, EDTA plasma, and saliva samples collected over a 72 h period to investigate the acute inflammatory response induced by LPS administration. Of the 130 saliva samples collected, 16 (two pigs per LPS dose, 0.5–3 h post-infusion) were selected for cytokine analysis, as this period was expected to show the greatest cytokine response. The aim was to evaluate whether saliva could serve as a potential matrix for cytokine measurement. Serum (*n* = 10 pigs; 13 time points), plasma (*n* = 10 pigs; 13 time points) and saliva samples (*n* = 4 pigs; 4 time points) were analyzed for the following cytokines: IL-1β, IL-6, IL-10 and IFN-γ via Luminex Discovery Assay, Porcine Premixed Multi-Analyte Kit (R&D Systems, Minneapolis, MN, USA) using the instrument MAGPIX ^®^ System according to manufacturer’s protocol. In addition, serum (*n* = 10 pigs; 13 time points) and saliva samples (*n* = 4 pigs; 4 time points) were analyzed for the following cytokines: IL-1α, IL-1ra, IL-2, IL-4, IL-8, IL-12, IL-18, GM-CSF and TNF-α via Luminex Discovery Assay, Porcine Premixed Multi-Analyte Kit (R&D Systems, Minneapolis, MN, USA) using the instrument MAGPIX^®^ System according to manufacturer’s protocol. All assays were performed in technical duplicates, and the mean value of the two measurements was used for statistical analysis. Blood samples were collected and processed individually for each pig. Intra- and inter-assay variation were in all instances below 8 and 10 CV%, respectively. Furthermore, blood samples were collected and analyzed individually for each pig.

### 2.4. Statistical Analysis

Statistical analyses were conducted in R, version 4.4.1. For all cytokines analyzed in serum and plasma, models were estimated using the glmmTMB package. The DHARMa package was used for model diagnostics and *p*-values were extracted using the emmeans package. To examine the impact of time on cytokines, we fitted generalized linear mixed models with a Zero Inflated Gamma distribution and log link (estimated using REML and the nlminb optimizer). The model included Time as fixed factor effect, Pig as a random effect and LPS dose per pig as a covariate. One observation point for the cytokines IL-6, IFN-γ, IL-10 and IL-1β was missing for one pig in the LOW LPS group at 0.5 h after LPS infusion due to a lost serum sample. Correlations were performed using Pearson correlation via the stats package. Measurements below the lower limit of quantification (LLOQ) were excluded from the correlation analyses. Statistical significance was defined as *p* ≤ 0.05, with *p* ≤ 0.10 considered indicative of a trend. Multiplicity adjustment of *p*-values was performed using the multivariate t (mvt) method in emmeans. For comparison of cytokine levels in saliva and serum, individual values for the same four pigs are presented as raw data.

## 3. Results

### 3.1. Cytokine Response in Plasma and Serum

**Figure 2:**
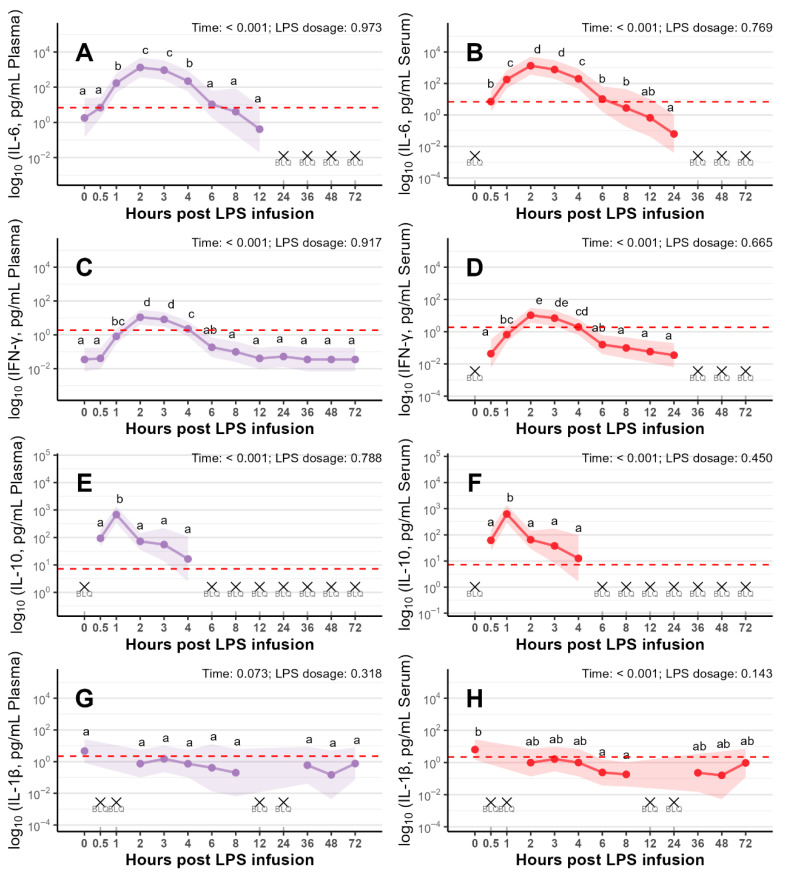
Concentrations (pg/mL) of the following cytokines in plasma and serum: IL-6 (plasma; (**A**)), IL-6 (serum; (**B**)), IFN-γ (plasma; (**C**)), IFN-γ (serum; (**D**)), IL-10 (plasma; (**E**)), IL-10 (serum; (**F**)), IL-1β (plasma; (**G**)) and IL-1β (serum; (**H**)) before (0 h) and up to 72 h after LPS infusion in pigs challenged with LOW or MODERATE LPS dose. Values are presented as marginal means with 95% confidence intervals. The lower limit of quantification (LLOQ) is indicated by a red dotted line. BLQ: all samples below LLOQ (not estimated). abcd: groups sharing a common letter do not differ (α = 0.05, mvt-adjusted). Sample sizes were *n* = 10 at all time points, except for serum at 0.5 h (*n* = 9). Data from LOW and MODERATE LPS doses were pooled (*n* = 10) to illustrate the overall temporal dynamics of cytokine concentrations.

The four cytokines (IL-6, IFN-γ, IL-10 and IL-1β) analyzed in plasma and serum showed similar temporal responses following LPS infusion (Figure 2A–H).

**Figure 3:**
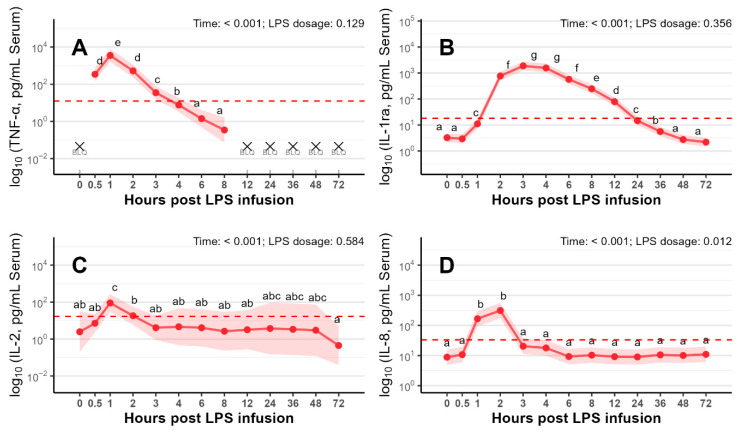
Concentrations (pg/mL) of the following cytokines in serum: TNF-α (**A**), IL-1ra (**B**), IL-2 (**C**) and IL-8 (**D**) before (0 h) and up to 72 h after LPS infusion in pigs challenged with LOW or MODERATE LPS dose. Values are presented as marginal means with 95% confidence intervals. The lower limit of quantification (LLOQ) is indicated by a red dotted line. BLQ: all samples below LLOQ (not estimated). abcdefg: groups sharing a common letter do not differ (α = 0.05, mvt-adjusted). Sample sizes were *n* = 10 at all time points. Data from LOW and MODERATE LPS doses were pooled (*n* = 10) to illustrate the overall temporal dynamics of cytokine concentrations.

The remaining 9 analyzed cytokines (IL-1α, IL-1ra, IL-2, IL-4, IL-8, IL-12, IL-18, GM-CSF, and TNF-α) in serum displayed distinct temporal responses following LPS infusion (Figure 3A–D and Figure S1A–E). In addition, descriptive statistics of all 13 serum cytokine concentrations (mean ± SD, range) are presented in Tables S1–S13. Most cytokines reached their peak concentrations 1 to 3 h following LPS infusion (*p* < 0.001). Particularly, TNF-α, IL-2 (Figure 3A and Figure 3C, respectively) and IL-10 (Figure 2F) exhibited a sharp and pronounced peak at 1 h after LPS administration. In contrast, IFN-γ, IL-6 (Figure 2D and Figure 2B, respectively) and IL-8 (Figure 3D) peaked between 2 and 3 h, while IL-1ra reached its peak response between 3 and 4 h post LPS infusion (Figure 3B).

### 3.2. Correlation Between Serum and Plasma Cytokines

**Figure 4:**
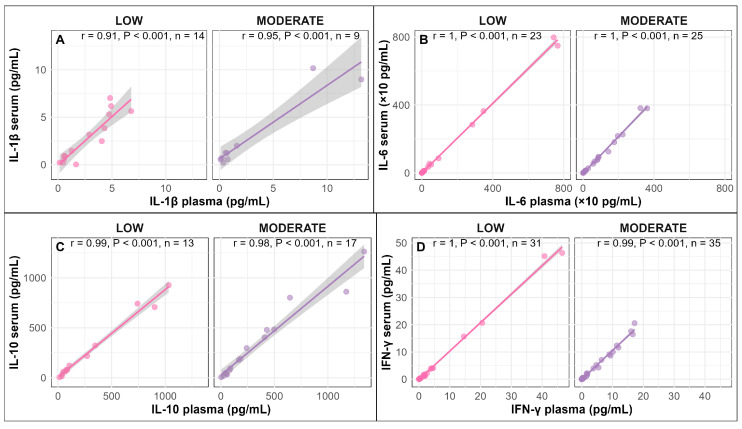
Correlations between serum and plasma for the cytokines IL-1β (**A**), IL-6, (**B**) IL-10 (**C**) and IFN-γ (**D**) in pigs infused with LOW or MODERATE LPS.

For the cytokines IL-1β, IL-6, IL-10 and IFN-γ, a strong positive correlation was observed between serum and plasma in pigs infused with LOW or MODERATE LPS (Pearson correlation coefficient: r = 0.91–1.00, *p* < 0.001; Figure 4A–D).

### 3.3. Correlation Between Serum Cytokines

**Figure 5:**
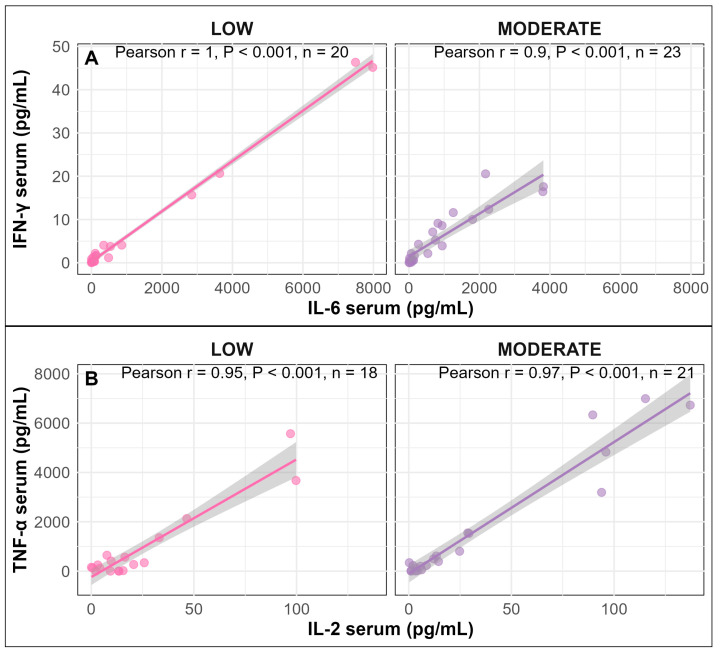
Positive correlations between IFN-γ and IL-6 (**A**) and between TNF-α and IL-2 (**B**) in serum of pigs infused with LOW or MODERATE LPS.

Moreover, in serum, a strong positive correlation was observed between IFN-γ and IL-6 (Pearson correlation coefficient: r = 0.90–1.00, *p* < 0.001; Figure 5A) as well as between TNF-α and IL-2 in pigs infused with LOW or MODERATE LPS dose (Pearson correlation coefficient: r = 0.95–0.97, *p* < 0.001; Figure 5B).

**Figure 6:**
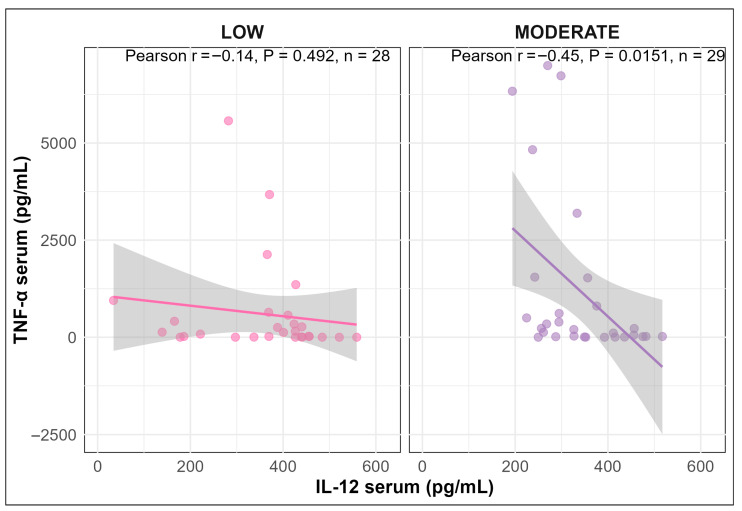
Correlations between TNF-α and IL-12 in serum of pigs infused with LOW or MODERATE LPS.

A negative correlation was observed between TNF-α and IL-12 in serum of pigs infused with LOW or MODERATE LPS; however, the correlation was only statistically significant for the MODERATE LPS group (Pearson correlation coefficient: r = −0.14, *p* = 0.492 for LOW; r = −0.45 *p* = 0.015 for MODERATE LPS; Figure 6).

### 3.4. Serum vs. Salivary Cytokines

**Figure 7:**
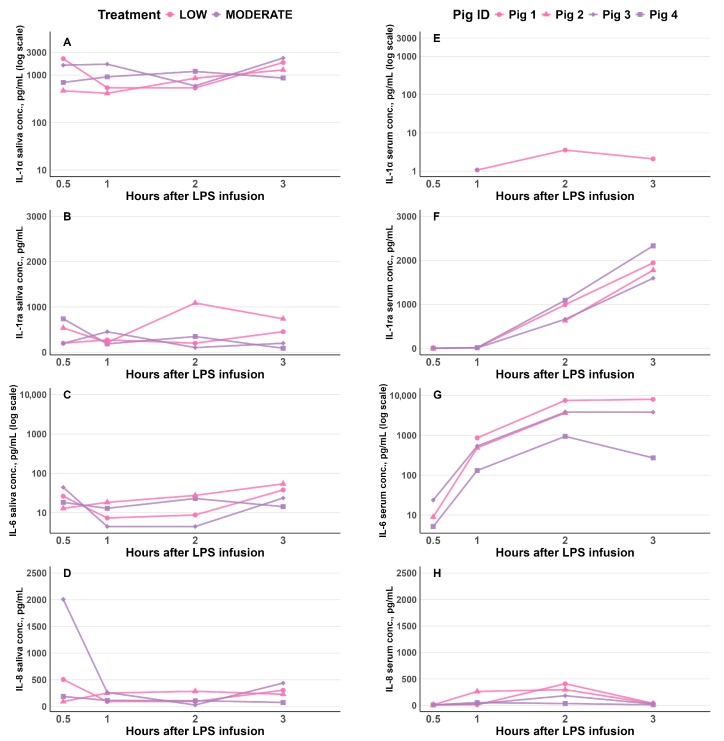
Salivary (**A**–**D**) and serum (**E**–**H**) concentrations (pg/mL) of IL-1α (**A**,**E**), IL-1ra (**B**,**F**), IL-6 (**C**,**G**), and IL-8 (**D**,**H**) in two pigs per LPS group (four pigs total) at 0.5, 1, 2 and 3 h after LPS infusion. Individual values represent raw data. In Figure 7G for serum IL-6 concentrations, one blood sample from Pig 1 was missing at 0.5 h post-infusion, resulting in a missing data point at this time point. Furthermore, serum IL-6 concentrations for Pig 2 at 3 h post LPS infusion were below the assay detection limit; thus no data point was shown for this observation.

Among the 13 cytokines analyzed in saliva samples, only IL-1α, IL-1ra, IL-6 and IL-8 were detectable (Figure 7A–D). In serum, for the corresponding pigs (Figure 7E–H), IL-1α was detectable in a few samples at 1, 2, and 3 h post LPS infusion, with lower concentrations than in saliva (Figure 7E and Figure 7A, respectively). IL-8 was detectable in most serum samples and was generally present at concentrations comparable to those in saliva; however, IL-8 serum concentrations appeared lower than those in saliva at 0.5 and 3 h following LPS infusion for the same four pigs (Figure 7H and Figure 7D, respectively). In contrast, IL-1ra were detectable in most serum samples, with concentrations comparable to or higher than those observed in saliva (Figure 7F and Figure 7B, respectively). Similarly, IL-6 was present in most serum samples at concentrations equal to or higher than those in saliva (Figure 7G and Figure 7C, respectively). Moreover, the serum concentrations of IL-1α, IL-1ra, and IL-6 appeared to follow a different trajectory compared to those in saliva.

### 3.5. Correlation Between Serum and Salivary Cytokines

**Figure 8:**
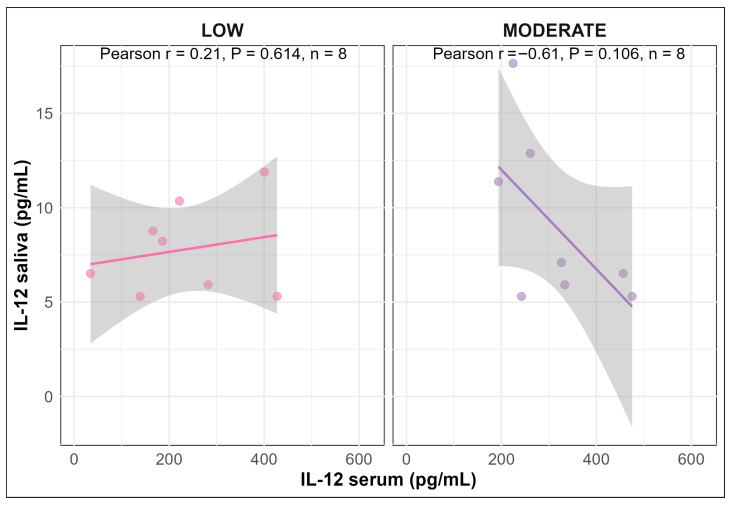
Correlations between saliva and serum for IL-12 in pigs infused with LOW or MODERATE LPS, respectively.

Overall, no significant correlations were observed between serum and saliva for any of the cytokines (Figure S2). However, for IL-12, a negative correlation was observed between serum and saliva in pigs infused with MODERATE LPS, although it was only a tendency (Pearson correlation coefficient: r = −0.61, *p* = 0.106; Figure 8). In contrast, pigs infused with LOW LPS showed a weak positive correlation between serum and saliva, which was not statistically significant (Pearson correlation coefficient: r = 0.21, *p* = 0.614; Figure 8).

## 4. Discussion

The early and pronounced peak of TNF-α, IL-2, and IL-10 at 1 h post LPS infusion reflects their roles as rapid responders during the early phase of the inflammatory response [15,22]. In contrast, IFN-γ and IL-6 peaked slightly later, between 2 and 3 h, consistent with other LPS studies in pigs, which may indicate their involvement in secondary immune activation processes [17,23]. For IL-6, the peak between 2 and 3 h is consistent with its function as a pleiotropic cytokine involved in both pro-inflammatory signaling and the transition to the anti-inflammatory phase by promoting acute phase protein synthesis and T cell differentiation [24,25]. The later peak of IL-1ra, occurring between 3 and 4 h post-infusion, might reflect its function as an anti-inflammatory mediator that helps regulate and resolve the inflammatory response [26]. Overall, these findings emphasize the coordinated and sequential nature of cytokine release following endotoxin challenge. Considering the peak responses of the cytokines, TNF-α appeared to be the most reliable cytokine to measure at 1 h during an acute inflammatory response, such as that induced by LPS, whereas IL-6 seemed to be most reliable for measurement between 2 and 3 h post LPS infusion. Thus, TNF-α serves as a reliable early biomarker of acute immune activation, optimal for detection within the first hour post-exposure, while IL-6 offers a slightly broader window for monitoring systemic inflammation, particularly between 2 and 3 h post-LPS challenge.

In general, a strong correlation between cytokine levels in serum and plasma have been well documented in humans [27,28,29,30,31]; however, comparable data in pigs are limited. In this study, IL-1β, IL-6, IL-10 and IFN-γ showed similar temporal responses and a strong positive correlation between serum and plasma, indicating that both serum and EDTA plasma provide reliable measurements of cytokine levels in blood of juvenile pigs. The absence of anticoagulants and the lower protein content in serum reduce potential interference with the analytical procedures used to measure blood components [32]. In addition, the use of serum samples may ease on-field handling of blood samples, as most serum samples can be left for 30 to 60 min before further processing.

Given that the animals in the present study were 12-week-old pigs and their immune systems were not yet fully mature, the composition of plasma or serum may differ from that of adult pigs, particularly under inflammatory conditions. Age-related variation could influence baseline cytokine levels as well as the intensity of cytokine responses. Future comparative studies in mature pigs will be valuable for assessing the consistency of cytokine-related responses across ages.

Furthermore, we observed strong positive correlations between IFN-γ and IL-6 as well as between TNF-α and IL-2 in serum of pigs infused with either LOW or MODERATE LPS, suggesting a coordinated immune response between these pro-inflammatory cytokines during exposure to *E. coli* LPS. Although the primary aim of this study was not to examine dose-dependent effects of LPS, the inclusion of two doses (LOW: 0.75 µg LPS/kg BW; MODERATE: 1.50 µg LPS/kg BW) was intended to mimic varying degrees of acute systemic inflammation, as previously described [20]. While cytokine kinetics were overall comparable between doses, subtle differences, such as stronger negative correlations between TNF-α and IL-12 in the MODERATE LPS group, may reflect nuanced immunomodulatory effects at higher endotoxin exposure. These observations underscore the importance of LPS dose when interpreting cytokine dynamics, particularly in translational research where precise modeling of inflammatory severity is critical. A negative correlation between TNF-α and IL-12 levels was observed in the serum, suggesting a potential regulatory interaction between these cytokines. This finding may indicate that TNF-α inhibits IL-12 production by macrophages, possibly as a mechanism to modulate the inflammatory response, similar to what has been observed in humans [33].

Relatively few studies have investigated cytokine concentrations in saliva from human or other species, particularly in relation to systemic inflammatory responses [18,19,34]. Most existing research on cytokine profiling in pigs has focused on blood [15,16] or tissue samples [35,36], with saliva remaining largely unexplored. It is well known that salivary glands secrete water, electrolytes, mucins, and a variety of proteins and glycoproteins that contribute to digestion, oral cavity cleansing and mucosal protection [37,38]. In addition to these primary components, saliva also contains bioactive molecules, including peptides and proteins involved in growth promotion, wound healing and regulation of immune and inflammatory responses, such as cytokines [39]. In the present study, 13 cytokines were analyzed in saliva samples between 0.5 and 3 h post-infusion. These time points were selected for analysis, as the 0.5 to 3 h post-infusion interval was expected to capture the strongest cytokine response and, consequently, the highest correlation with serum concentrations. Of the 13 cytokines, IL-1α, IL-1ra, IL-6 and IL-8 were detectable in saliva. Interestingly, some cytokines, including IL-1α and IL-8, were present at higher concentrations in saliva than in blood. This suggests an active role for the oral mucosal-salivary gland axis in cytokine production and secretion, rather than passive translocation from plasma.

Llamas Moya, Boyle, Lynch and Arkins [18] reported detectable TNF-α and IL-1β in saliva following LPS injection in 6-week-old mixed-sex pigs with higher concentrations in saliva than in plasma. This contrasts with our findings, as TNF-α was undetectable in saliva despite a pronounced peak in serum, while IL-1β was detected at low concentrations in serum and remained undetectable in saliva. Consistent with our findings, Huang, Liu, Yin, Ci, Zhao and Yang [34] reported undetectable salivary TNF-α concentrations in male pigs (average BW 12 kg) after LPS injection, despite elevated levels in plasma. However, certain cytokines in the current study, specifically IL-1α and IL-8, showed higher concentrations in saliva than in serum. Since IL-1α was detected at higher concentrations in saliva than in serum at all measured time points, this may suggest that saliva might serve as a suitable matrix for assessing selected cytokines in response to LPS-induced inflammation.

Yao, Li, Murdiastuti, Kosugi-Tanaka, Akamatsu, Kanamori and Hosoi [39] reported that intraperitoneal injection of LPS in mice induced upregulation of IL-1β, IL-6 and TNF-α expression via TLR-4 signaling in the submandibular glands, followed by secretion of these cytokines into saliva. Their finding suggests that certain cytokines can be locally produced in salivary glands in response to LPS stimulation in mice and potentially in pigs; however, research on this subject in pigs remains limited. In addition to local production of cytokines, salivary glands are highly permeable and closely associated with capillary networks that enable molecular exchange [40], which may also be the cause for the observed negative correlation between salivary and serum IL-12 concentrations. Furthermore, IL-1ra, IL-6 and IL-8 were detected at high concentrations in serum and were also detectable in saliva, which may suggest that these cytokines were transferred from the bloodstream into saliva. However, the current study could not determine whether the cytokines detected in saliva originated from local production within the salivary glands or were transferred from the blood circulation.

The dynamics of the salivary cytokine response did not correspond to those of the serum response upon acute inflammation. However, in the current study, only a limited number of saliva samples were analyzed for cytokine concentrations. Thus, further research is needed to elucidate the origin of salivary cytokines following LPS stimulation and to clarify the relationship between cytokine concentrations in saliva and blood in pigs.

## 5. Conclusions

TNF-α and IL-6 appear to be the most reliable cytokines for monitoring acute inflammatory responses in pigs, peaking at 1 h and between 2 and 3 h post-infusion, respectively. Furthermore, the results indicate that both serum and EDTA plasma are suitable for cytokine quantification in pig blood upon acute inflammation; however, serum may offer practical advantages by facilitating blood sample handling. Although IL-1α, IL-1ra, IL-6 and IL-8 were detectable in saliva shortly after LPS infusion, saliva did not correspond to serum cytokine levels in a porcine model of acute inflammation. However, IL-1α was detected at higher concentrations in saliva than in serum, suggesting that saliva may serve as a suitable matrix for assessing selected cytokines in response to LPS-induced inflammation. Further research is, however, needed to investigate the origin of salivary cytokines and their role upon LPS stimulation.

## Data Availability

The original contributions presented in this study are included in the article/Supplementary Material. Further inquiries can be directed to the corresponding author.

## Supplementary Materials

The following supporting information can be downloaded at: https://www.mdpi.com/article/10.3390/vetsci13010068/s1, Figure S1: Serum concentration (pg/mL) of the following cytokines IL-4 (A), IL-12 (B), IL-18 (C), IL-1α (D) and GMCSF (E) in pigs before and after LOW or MODERATE LPS. Figure S2: Correlations between saliva and serum for IL-1α, IL-1ra, IL-2, IL-4, IL-6, IL-8, IL-10, TNF-α, IFN-γ, GM-CS, IL-1β and IL-18 in pigs infused with LOW or MODERATE LPS dose. Tables S1–S13 present the descriptive statistics (mean ± SD, range) of serum cytokine concentrations following LPS infusion in pigs. The cytokines included are IFN-γ, TNF-α, IL-1α, IL-1β, IL-1ra, IL-2, IL-4, IL-6, IL-8, IL-10, IL-12, IL-18, and GM-CSF.

## Abbreviations

The following abbreviations are used in this manuscript:

GM-CSF	Granulocyte macrophage colony stimulating factor
IFN-γ	Interferon gamma
IL-1α	Interleukin 1 alpha
IL-1β	Interleukin 1 beta
IL-1ra	Interleukin 1 receptor antagonist
IL-2	Interleukin 2
IL-4	Interleukin 4
IL-6	Interleukin 6
IL-8	Interleukin 8
IL-10	Interleukin 10
IL-12	Interleukin 12
IL-18	Interleukin 18
LLOQ	Lower limit of quantification
LPS	Lipopolysaccharide
TNF-α	Tumor necrosis factor alpha
