# Supplementary material for: Comparative Study of Cytokine Measurements in Blood Plasma and Serum, and Saliva of Juvenile Pigs During Experimentally Induced Acute Inflammation
Source: Vet Sci. 2026 Jan 9;13(1):68. doi: 10.3390/vetsci13010068 (PMC12846541; doi:10.3390/vetsci13010068)
Supplement: Supplementary file 1 [file vetsci-13-00068-s001.zip › vetsci-3987389-supplementary.pdf]

Supplementary material

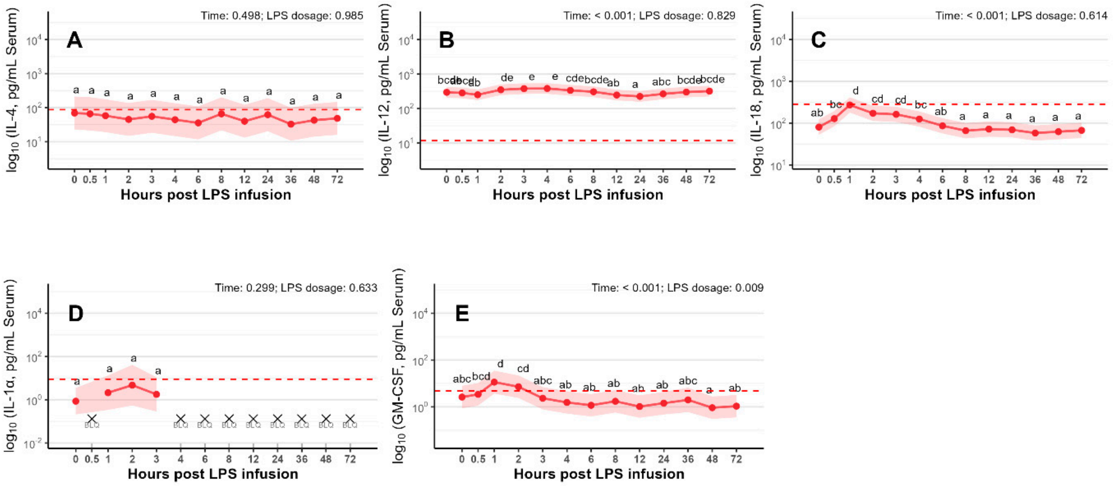

Figure S1. Serum concentration (pg/mL) of the following cytokines IL-4 (A), IL-12 (B), IL-18 (C), IL-1α (D) and GMCSF (E) in pigs before and after LOW or MODERATE LPS. Values are presented as marginal means along with 95% confidence interval. Lower limit of quantification is illustrated as a red dotted line. The lower limit of quantification (LOQ) is indicated by a red dotted line. BLQ: all samples below LOQ (not estimated). abc: groups sharing a common letter do not differ (α = 0.05, mtv-adjusted). Sample sizes were n = 10 at all time points.

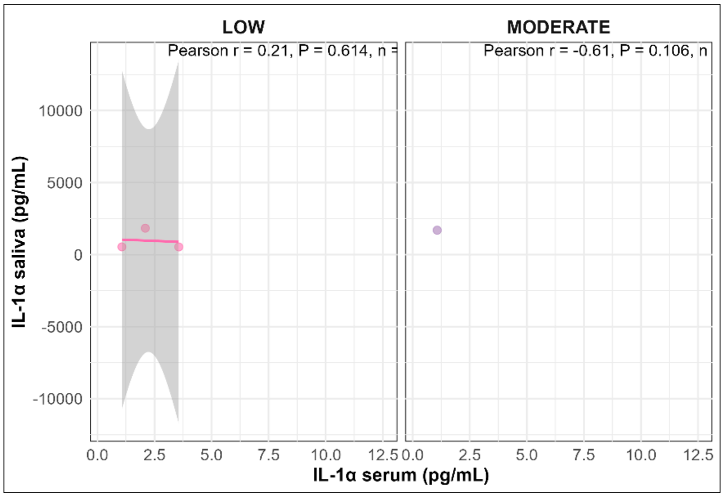
LOW
MODERATE
Pearson r = 0.21, P = 0.614, n =
Pearson r = -0.61, P = 0.106, n
IL-1α saliva (pg/mL)
10000
5000
0
-5000
-10000
0.0
2.5
5.0
7.5
10.0
12.5
IL-1α serum (pg/mL)

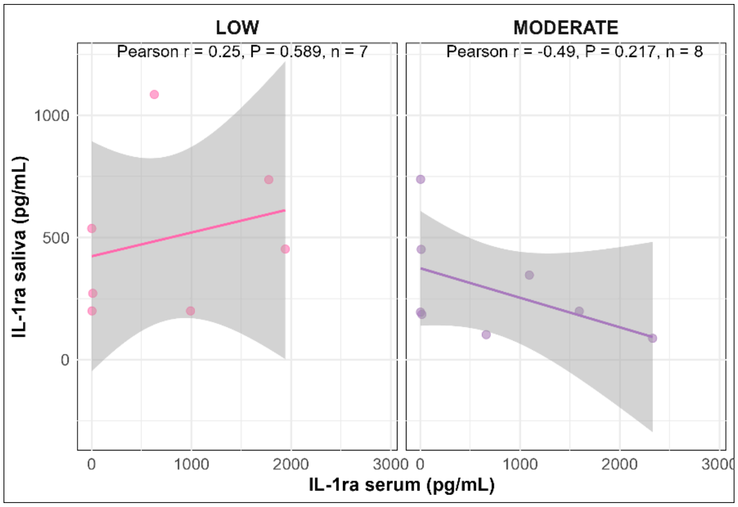
LOW
MODERATE
Pearson r = 0.25, P = 0.589, n = 7
Pearson r = -0.49, P = 0.217, n = 8
IL-1ra saliva (pg/mL)
1000
500
0
0
1000
2000
3000
IL-1ra serum (pg/mL)

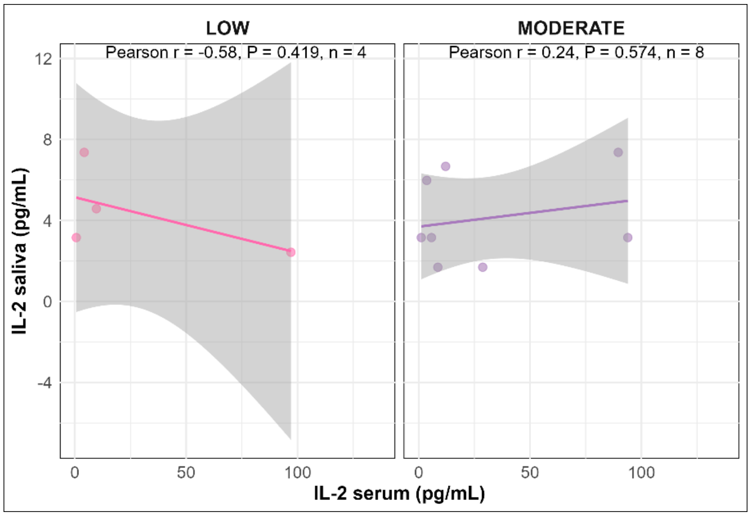
LOW
MODERATE
Pearson r = -0.58, P = 0.419, n = 4
Pearson r = 0.24, P = 0.574, n = 8
IL-2 saliva (pg/mL)
12
8
4
0
-4
0
50
100
IL-2 serum (pg/mL)

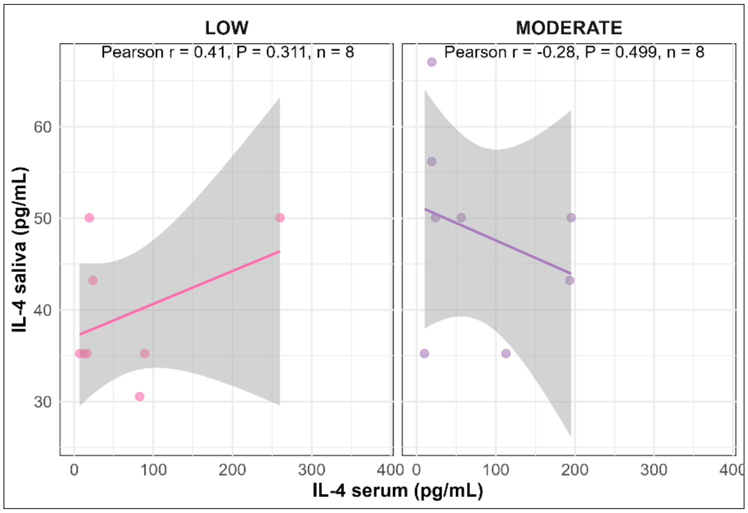
LOW
MODERATE
Pearson r = 0.41, P = 0.311, n = 8
Pearson r = -0.28, P = 0.499, n = 8
IL-4 saliva (pg/mL)
IL-4 serum (pg/mL)

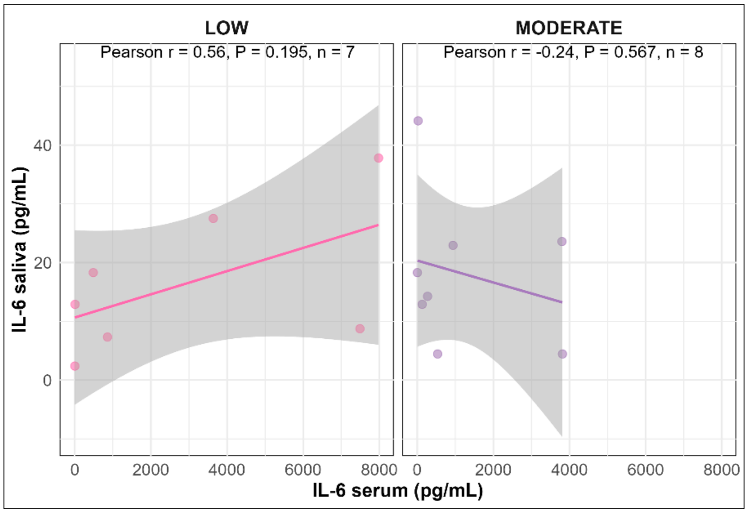
LOW
MODERATE
Pearson r = 0.56, P = 0.195, n = 7
Pearson r = -0.24, P = 0.567, n = 8
IL-6 saliva (pg/mL)
IL-6 serum (pg/mL)

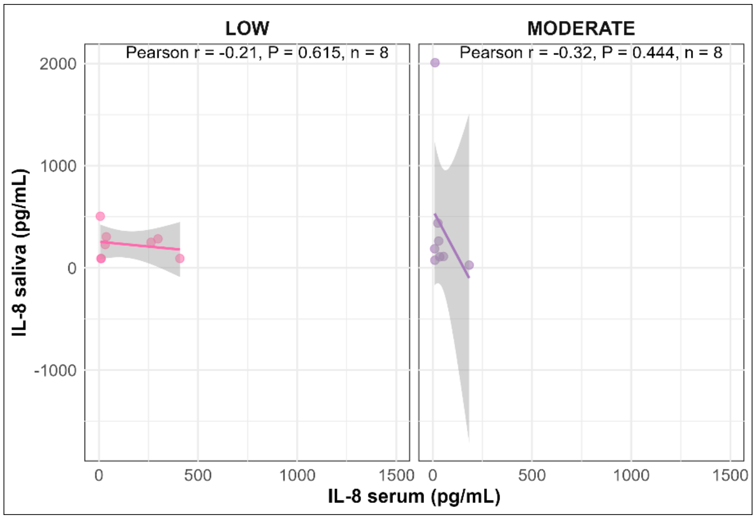
LOW
MODERATE
Pearson r = -0.21, P = 0.615, n = 8
Pearson r = -0.32, P = 0.444, n = 8
IL-8 saliva (pg/mL)
IL-8 serum (pg/mL)

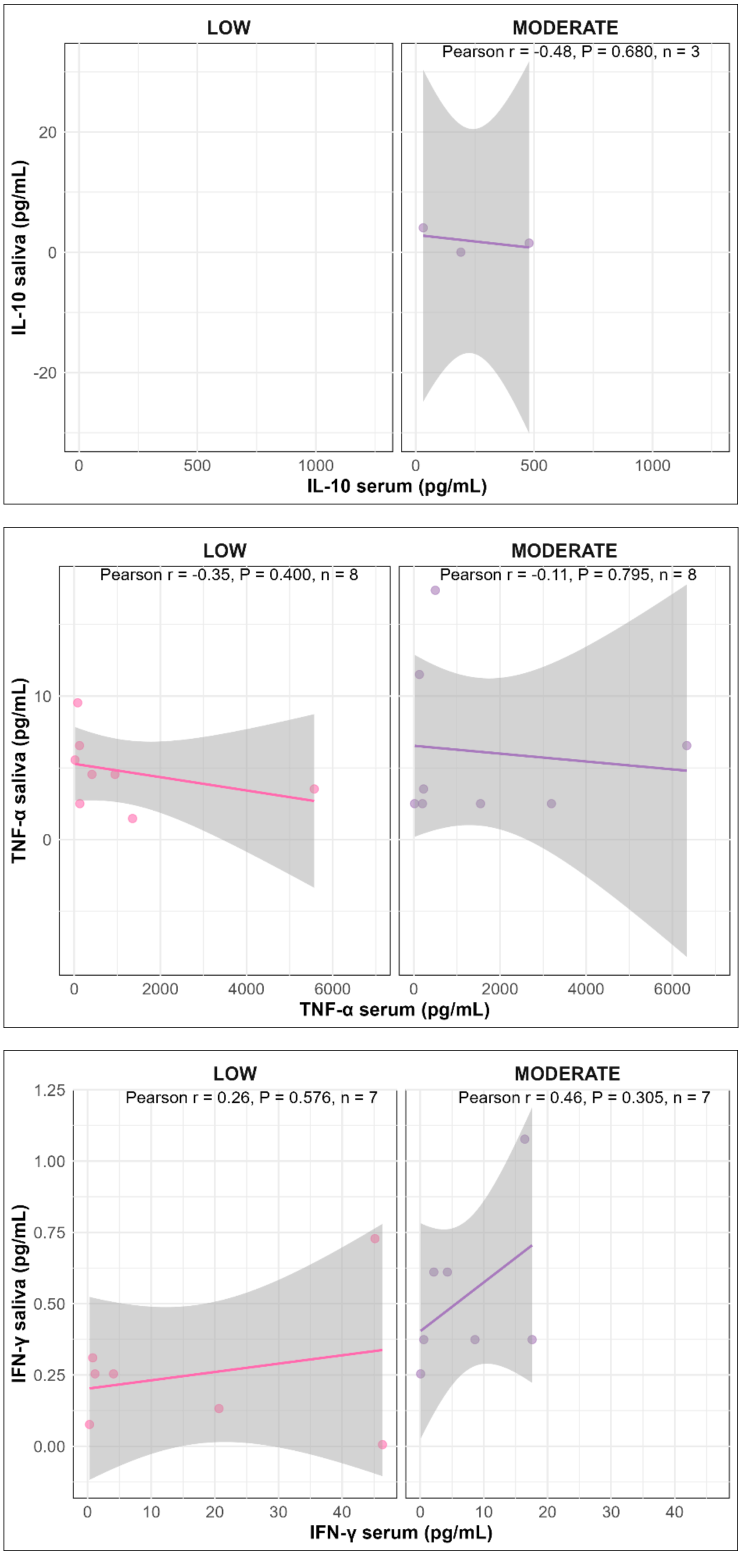
LOW
MODERATE
Pearson r = -0.48, P = 0.680, n = 3
IL-10 saliva (pg/mL)
IL-10 serum (pg/mL)
20
0
-20
0
500
1000
LOW
MODERATE
Pearson r = -0.35, P = 0.400, n = 8
Pearson r = -0.11, P = 0.795, n = 8
TNF-α saliva (pg/mL)
TNF-α serum (pg/mL)
10
0
0
2000
4000
6000
LOW
MODERATE
Pearson r = 0.26, P = 0.576, n = 7
Pearson r = 0.46, P = 0.305, n = 7
IFN-γ saliva (pg/mL)
IFN-γ serum (pg/mL)
1.25
1.00
0.75
0.50
0.25
0.00
0
10
20
30
40

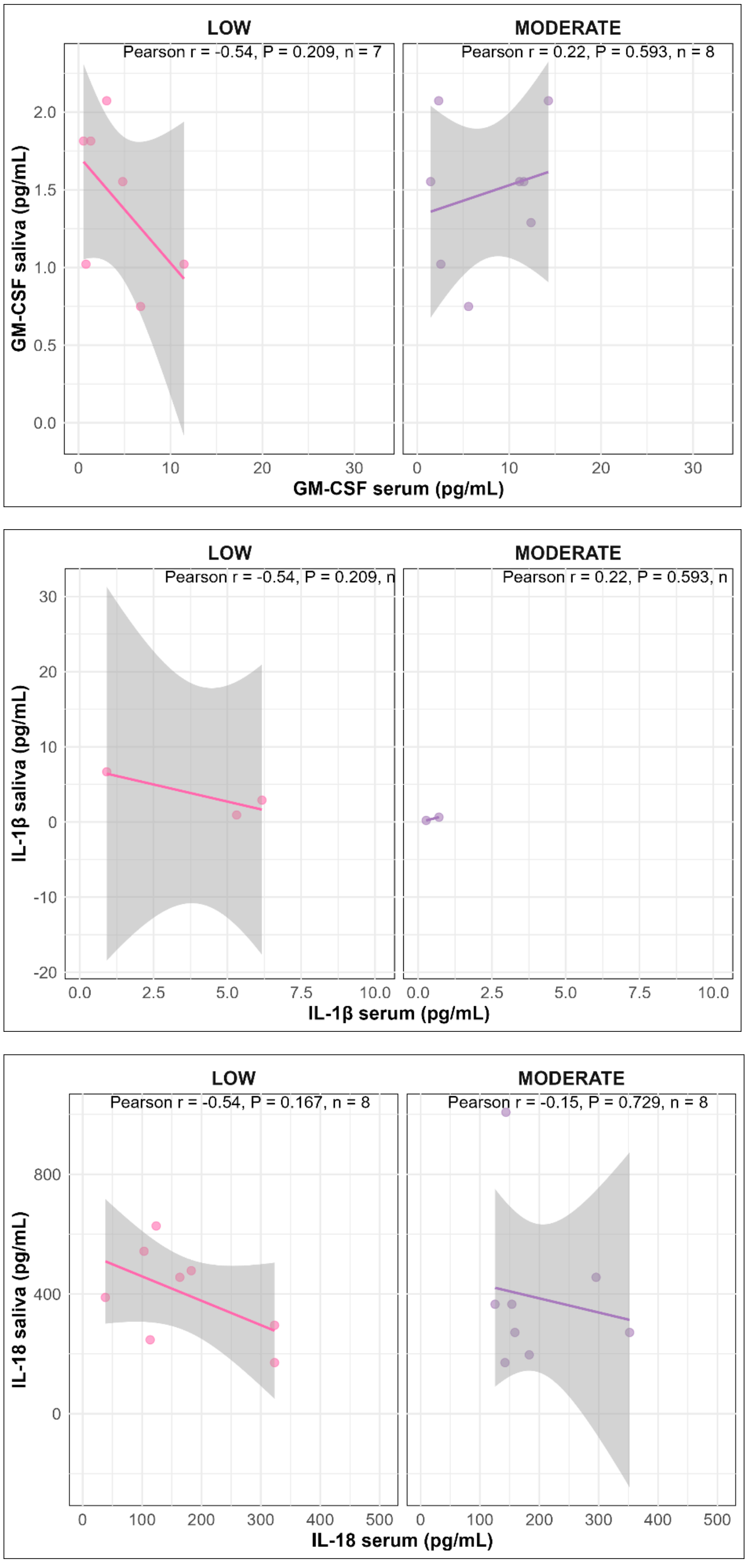

Figure S2. Correlations between saliva and serum for IL-1α, IL-1ra, IL-2, IL-4, IL-6, IL-8, IL-10, TNF-α, IFN-γ, GM-CS, IL-1β and IL-18 in pigs infused with LOW or MODERATE LPS dose.

Table S1. Descriptive statistics of serum IFN-γ concentrations (pg/mL) following LPS infusion in pigs (mean ± SD, range).

LPS dose (µg/kg BW)	Time (h after LPS infusion)	Mean (pg/mL)	SD (pg/mL)	Range (pg/mL)
0.75	0	0.049	0.039	0.021–0.076
1.50	0	0.133	–	0.133
0.75	0.5	–	–	–
1.50	0.5	0.046	0.043	0.015–0.076
0.75	1	1.235	1.632	0.308–4.078
1.50	1	0.743	0.802	0.113–2.144
0.75	2	15.40	18.86	2.179–46.33
1.50	2	13.18	5.505	8.615–20.56
0.75	3	12.24	21.95	0.813–45.16
1.50	3	10.36	4.744	4.283–16.44
0.75	4	4.152	7.685	0.308–15.68
1.50	4	2.784	1.734	1.051–5.219
0.75	6	0.794	1.015	0.076–1.512
1.50	6	0.140	0.259	0.001–0.528
0.75	8	0.193	0.165	0.076–0.310
1.50	8	0.105	0.040	0.076–0.133
0.75	12	0.049	0.039	0.021–0.076
1.50	12	0.046	0.043	0.015–0.076
0.75	24	0.076	–	0.076
1.50	24	0.046	0.043	0.015–0.076
0.75	36	0.076	–	0.076
1.50	36	0.046	0.043	0.015–0.076
0.75	48	0.076	–	0.076
1.50	48	0.015	–	0.015
0.75	72	0.076	–	0.076
1.50	72	0.015	–	0.015

Table S2. Descriptive statistics of serum TNF-α concentrations (pg/mL) following LPS infusion in pigs (mean ± SD, range).

LPS dose (µg/kg BW)	Time (h after LPS infusion)	Mean (pg/mL)	SD (pg/mL)	Range (pg/mL)
0.75	0	–	–	–
1.50	0	–	–	–
0.75	0.5	259.99	189.01	81.42–567.09
1.50	0.5	331.47	122.18	198.68–498.02
0.75	1	2593.31	2046.88	643.41–5571.41
1.50	1	5616.01	1593.09	3192.66–6994.20
0.75	2	457.55	524.14	25.42–1354.78
1.50	2	943.69	581.13	225.49–1547.71
0.75	3	34.54	50.69	1.86–124.09

1.50	3	67.54	48.9	16.30–128.35
0.75	4	9.07	10.55	1.22–21.06
1.50	4	13.34	7.97	4.09–21.93
0.75	6	2.09	2.72	0.16–4.01
1.50	6	3.05	2.09	1.22–5.33
0.75	8	1.22	–	1.22
1.50	8	0.16	–	0.16
0.75	12	–	–	–
1.50	12	–	–	–
0.75	24	–	–	–
1.50	24	–	–	–
0.75	36	0.16	–	0.16
1.50	36	–	–	–
0.75	48	–	–	–
1.50	48	–	–	–
0.75	72	0.16	–	0.16
1.50	72	–	–	–

Table S3. Descriptive statistics of serum IL-1α concentrations (pg/mL) following LPS infusion in pigs (mean ± SD, range).

LPS dose (μg/kg BW)	Time (h after LPS infusion)	Mean (pg/mL)	SD (pg/mL)	Range (pg/mL)
0.75	0	0.652	0.168	0.476–0.811
1.50	0	3.767	4.924	0.286–7.248
0.75	0.5	–	–	–
1.50	0.5	–	–	–
0.75	1	1.073	–	1.073
1.50	1	1.436	0.514	1.073–1.799
0.75	2	3.563	–	3.563
1.50	2	–	–	–
0.75	3	2.097	–	2.097
1.50	3	0.492	–	0.492
0.75	4	–	–	–
1.50	4	–	–	–
0.75	6	–	–	–
1.50	6	–	–	–
0.75	8	–	–	–
1.50	8	–	–	–
0.75	12	–	–	–
1.50	12	–	–	–
0.75	24	–	–	–
1.50	24	0.674	–	0.674
0.75	36	9.006	–	9.006
1.50	36	–	–	–

0.75	48	0.811	–	0.811
1.50	48	–	–	–
0.75	72	12.521	–	12.521
1.50	72	0.811	–	0.811

Table S4. Descriptive statistics of serum IL-1β concentrations (pg/mL) following LPS infusion in pigs (mean ± SD, range).

LPS dose (µg/kg BW)	Time (h after LPS infusion)	Mean (pg/mL)	SD (pg/mL)	Range (pg/mL)
0.75	0	5.5	1.59	3.84–7.02
1.50	0	6.8	4.84	1.26–10.16
0.75	0.5	–	–	–
1.50	0.5	–	–	–
0.75	1	–	–	–
1.50	1	–	–	–
0.75	2	3.11	3.11	0.91–5.31
1.50	2	0.57	–	0.57
0.75	3	6.17	–	6.17
1.50	3	1	0.9	0.27–2.01
0.75	4	3.18	–	3.18
1.50	4	0.73	0.72	0.22–1.23
0.75	6	1.47	–	1.47
1.50	6	0.14	0.2	0.004–0.37
0.75	8	0.94	–	0.94
1.50	8	0.08	0.07	0.003–0.15
0.75	12	0.15	–	0.15
1.50	12	0.05	–	0.05
0.75	24	–	–	–
1.50	24	–	–	–
0.75	36	0.31	0.41	0.026–0.60
1.50	36	–	–	–
0.75	48	0.22	–	0.22
1.50	48	–	–	–
0.75	72	1.35	1.6	0.22–2.48
1.50	72	0.55	–	0.55

Table S5. Descriptive statistics of serum IL-1ra concentrations (pg/mL) following LPS infusion in pigs (mean ± SD, range).

LPS dose (µg/kg BW)	Time (h after LPS infusion)	Mean (pg/mL)	SD (pg/mL)	Range (pg/mL)
0.75	0	3.82	2.1	1.49–6.49
1.50	0	2.84	1.3	1.35–4.02
0.75	0.5	3.3	1	1.91–4.46

1.50	0.5	2.67	0.75	2.08–3.69
0.75	1	10.58	2.19	7.78–12.56
1.50	1	12.53	3.7	9.06–17.03
0.75	2	702.67	211.87	447.58–992.36
1.50	2	884.26	315.19	463.98–1229.14
0.75	3	1645.29	386.16	1150.33–2027.78
1.50	3	2178.13	569.42	1593.80–2922.16
0.75	4	1252.73	322.14	886.44–1607.06
1.50	4	1932.14	585.07	1186.61–2781.69
0.75	6	503.19	302.04	230.61–984.84
1.50	6	709.21	286.87	342.81–1082.21
0.75	8	226.07	182.48	101.49–543.45
1.50	8	301.34	109.82	143.16–410.43
0.75	12	71.63	46.84	32.78–148.37
1.50	12	91.62	21.44	66.21–125.20
0.75	24	14.49	5.43	7.76–21.57
1.50	24	14.85	5.69	9.08–22.42
0.75	36	5.18	1.28	3.27–6.39
1.50	36	6.46	2.02	3.04–8.25
0.75	48	2.87	1.31	1.28–4.58
1.50	48	2.91	1.08	1.49–3.69
0.75	72	2.23	1.55	0.68–4.58
1.50	72	2.32	1.32	1.08–4.58

Table S6. Descriptive statistics of serum IL-2 concentrations (pg/mL) following LPS infusion in pigs (mean ± SD, range).

LPS dose (μg/kg BW)	Time (h after LPS infusion)	Mean (pg/mL)	SD (pg/mL)	Range (pg/mL)
0.75	0	13.45	–	13.45
1.50	0	0.08	–	0.081
0.75	0.5	10.21	9.8	0.64–20.66
1.50	0.5	6.93	6.18	0.276–14.48
0.75	1	62.73	44.14	7.64–99.73
1.50	1	106.4	19.79	89.63–137.10
0.75	2	17.17	15.03	0.081–33.11
1.50	2	20.98	9.43	8.59–29.35
0.75	3	9.86	7.99	4.21–15.51
1.50	3	3.67	2.55	1.15–6.25
0.75	4	13.45	–	13.45
1.50	4	4.19	–	4.19
0.75	6	13.45	–	13.45
1.50	6	3.17	–	3.17
0.75	8	11.39	–	11.39
1.50	8	1.11	–	1.11

0.75	12	14.48	–	14.48
1.50	12	1.11	–	1.11
0.75	24	10.36	–	10.36
1.50	24	–	–	–
0.75	36	9.33	–	9.33
1.50	36	–	–	–
0.75	48	8.3	–	8.3
1.50	48	–	–	–
0.75	72	2.14	–	2.14
1.50	72	0.08	–	0.081

Table S7. Descriptive statistics of serum IL-4 concentrations (pg/mL) following LPS infusion in pigs (mean ± SD, range).

LPS dose (µg/kg BW)	Time (h after LPS infusion)	Mean (pg/mL)	SD (pg/mL)	Range (pg/mL)
0.75	0	111.23	82.7	19.12–227.61
1.50	0	43.3	13.54	19.12–50.68
0.75	0.5	91.54	101.24	16.08–259.99
1.50	0.5	34.95	16.77	19.12–56.37
0.75	1	90.71	74.99	12.72–212.38
1.50	1	55.16	34.88	23.66–112.76
0.75	2	100.3	126.73	6.87–315.83
1.50	2	61.19	77.08	3.03–193.45
0.75	3	107.34	156.91	19.12–384.55
1.50	3	70.81	75.92	3.03–195.26
0.75	4	67.49	97.25	0.70–236.44
1.50	4	72.39	41.48	19.12–118.80
0.75	6	88.27	142.84	0.70–341.75
1.50	6	52.39	34.71	0.70–87.53
0.75	8	99.95	150.09	0.70–362.90
1.50	8	113.85	71.55	19.12–212.32
0.75	12	73.1	90.39	0.70–229.15
1.50	12	45.9	34.08	21.31–103.26
0.75	24	79.48	117.24	7.48–287.38
1.50	24	100.27	100.01	9.91–244.76
0.75	36	81.44	89.31	0.70–220.57
1.50	36	25.25	23.24	7.48–50.68
0.75	48	81.73	122.84	7.48–292.64
1.50	48	51.03	26.25	14.45–88.69
0.75	72	69.74	83.85	7.48–196.04
1.50	72	58.76	49.35	7.48–128.06

Table S8. Descriptive statistics of serum IL-6 concentrations (pg/mL) following LPS infusion in pigs (mean ± SD, range).

LPS dose (μg/kg BW)	Time (h after LPS infusion)	Mean (pg/mL)	SD (pg/mL)	Range (pg/mL)
0.75	0	–	–	–
1.50	0	–	–	–
0.75	0.5	6.056	3.935	1.564–8.896
1.50	0.5	9.694	12.289	0.328–23.61
0.75	1	316.32	351.72	66.603–860.08
1.50	1	184.07	199.13	68.176–536.89
0.75	2	2425.37	3176.03	110.26–7489.91
1.50	2	1911.4	1207.06	823.18–3814.09
0.75	3	2040.06	3959.42	2.396–7978.63
1.50	3	1654.21	1414.47	271.50–3800.15
0.75	4	1427.28	2005.91	8.896–2845.67
1.50	4	388.74	428.74	21.49–944.92
0.75	6	120.66	–	120.67
1.50	6	18.69	0.21	18.55–18.84
0.75	8	21.49	–	21.49
1.50	8	–	–	–
0.75	12	5.15	–	5.15
1.50	12	–	–	–
0.75	24	0.48	–	0.485
1.50	24	–	–	–
0.75	36	–	–	–
1.50	36	–	–	–
0.75	48	–	–	–
1.50	48	–	–	–
0.75	72	1.92	–	1.92
1.50	72	–	–	–

Table S9. Descriptive statistics of serum IL-8 concentrations (pg/mL) following LPS infusion in pigs (mean ± SD, range).

LPS dose (μg/kg BW)	Time (h after LPS infusion)	Mean (pg/mL)	SD (pg/mL)	Range (pg/mL)
0.75	0	8.39	1.54	6.49–10.71
1.50	0	9.16	3.46	5.64–13.73
0.75	0.5	8.22	2.18	5.28–10.41
1.50	0.5	13.85	9.88	7.09–31.27
0.75	1	109.32	135.37	7.29–262.88
1.50	1	241.15	247.33	29.33–539.74
0.75	2	193.07	167.07	9.58–408.48
1.50	2	469.08	592	34.69–1494.97
0.75	3	19.07	14.45	6.89–37.42

1.50	3	21.85	13.93	10.20–44.19
0.75	4	8.98	1.85	6.49–10.49
1.50	4	28.47	40.65	4.18–100.44
0.75	6	8.92	1.47	6.89–10.28
1.50	6	9.47	3.05	5.22–13.33
0.75	8	9.79	2.29	6.89–13.32
1.50	8	10.7	3.33	7.09–14.56
0.75	12	8.84	1.32	7.29–9.91
1.50	12	9.35	3.31	6.26–14.13
0.75	24	9.99	2.75	5.84–13.33
1.50	24	7.36	2.16	5.01–10.71
0.75	36	9.76	1.51	7.29–11.32
1.50	36	11.25	2.31	8.90–13.73
0.75	48	9.59	1.98	6.49–11.32
1.50	48	10.45	1.55	8.90–12.12
0.75	72	10.15	3.25	5.69–13.73
1.50	72	11.8	1.46	10.49–13.73

Table S10. Descriptive statistics of serum IL-10 concentrations (pg/mL) following LPS infusion in pigs (mean ± SD, range).

LPS dose (µg/kg BW)	Time (h after LPS infusion)	Mean (pg/mL)	SD (pg/mL)	Range (pg/mL)
0.75	0	–	–	–
1.50	0	–	–	–
0.75	0.5	69.1	59.5	4.97–122.4
1.50	0.5	120.9	121.7	16.17–296.5
0.75	1	583.2	300	217.8–926.4
1.50	1	763	341.8	405.1–1263.3
0.75	2	50.72	26.17	13.70–79.91
1.50	2	147.8	196.6	6.05–478.8
0.75	3	–	–	–
1.50	3	114.3	107.8	38.03–190.5
0.75	4	–	–	–
1.50	4	46.44	–	46.44
0.75	6	–	–	–
1.50	6	–	–	–
0.75	8	–	–	–
1.50	8	–	–	–
0.75	12	–	–	–
1.50	12	–	–	–
0.75	24	–	–	–
1.50	24	–	–	–
0.75	36	–	–	–
1.50	36	–	–	–

0.75	48	_	_	_
1.50	48	_	_	_
0.75	72	_	_	_
1.50	72	_	_	_

Table S11. Descriptive statistics of serum IL-12 concentrations (pg/mL) following LPS infusion in pigs (mean ± SD, range).

LPS dose (µg/kg BW)	Time (h after LPS infusion)	Mean (pg/mL)	SD (pg/mL)	Range (pg/mL)
0.75	0	342.73	151.17	144.08–561.21
1.50	0	277.69	44.04	205.70–326.11
0.75	0.5	319.96	132.03	139.12–440.52
1.50	0.5	273.97	38.54	224.69–326.49
0.75	1	284.36	144.69	34.33–370.70
1.50	1	266.76	53.95	193.98–333.48
0.75	2	379.61	120.25	165.73–455.12
1.50	2	345.17	81.57	242.26–456.62
0.75	3	393.41	119.82	185.99–484.32
1.50	3	386.15	90.36	260.96–475.38
0.75	4	372.28	112.58	178.05–441.34
1.50	4	410.33	94.09	287.50–517.37
0.75	6	368.38	143.59	140.45–521.20
1.50	6	338.93	68.4	249.78–435.74
0.75	8	336.8	140.42	109.79–455.91
1.50	8	316.28	71.29	199.03–392.51
0.75	12	286.32	124.78	98.42–414.69
1.50	12	237.54	36.69	193.60–291.21
0.75	24	241.13	117.23	79.56–371.32
1.50	24	247.55	115.47	126.29–415.08
0.75	36	350.17	183.3	103.69–572.70
1.50	36	232.37	56.56	144.19–295.47
0.75	48	387.43	208.06	124.87–622.77
1.50	48	269.28	66.63	161.19–333.54
0.75	72	364.33	161.56	155.67–558.75
1.50	72	304.59	73.92	225.35–402.61

Table S12. Descriptive statistics of serum IL-18 concentrations (pg/mL) following LPS infusion in pigs (mean ± SD, range).

LPS dose (µg/kg BW)	Time (h after LPS infusion)	Mean (pg/mL)	SD (pg/mL)	Range (pg/mL)
0.75	0	94.71	34.19	60.75–143.91
1.50	0	75.84	43.77	38.58–145.41
0.75	0.5	132.96	34.49	103.35–191.80

1.50	0.5	129.66	65.37	56.81–230.27
0.75	1	206.1	122.83	38.32–323.14
1.50	1	365.37	82.32	295.68–506.40
0.75	2	151.79	97.29	91.24–323.14
1.50	2	194.45	25.77	159.05–224.55
0.75	3	145.64	28.91	110.56–182.91
1.50	3	182.2	56.06	142.36–280.19
0.75	4	103.4	26.49	74.95–143.91
1.50	4	155.32	73.11	103.35–280.19
0.75	6	81.13	16.56	60.75–103.35
1.50	6	98.32	46.18	71.76–179.69
0.75	8	61.79	21.62	38.10–82.38
1.50	8	80.77	45.87	56.81–162.05
0.75	12	79.74	19.01	49.74–92.75
1.50	12	70.99	40.93	20.34–127.99
0.75	24	73.72	41.81	38.10–145.41
1.50	24	71.86	36	20.34–110.56
0.75	36	64.61	21.37	26.46–74.95
1.50	36	65.47	66.26	10.74–179.69
0.75	48	57.49	16.63	38.09–74.95
1.50	48	78.31	58.81	38.09–179.69
0.75	72	61.9	16.97	38.09–74.95
1.50	72	82	47.22	38.09–145.41

Table S13. Descriptive statistics of serum GM-CSF concentrations (pg/mL) following LPS infusion in pigs (mean ± SD, range).

LPS dose (μg/kg BW)	Time (h after LPS infusion)	Mean (pg/mL)	SD (pg/mL)	Range (pg/mL)
0.75	0	2.559	2.017	0.284–4.340
1.50	0	6.118	11.287	0.555–26.298
0.75	0.5	2.449	1.532	0.816–4.813
1.50	0.5	7.433	11.409	1.755–27.832
0.75	1	8.052	3.403	3.560–11.474
1.50	1	17.943	8.49	11.133–32.733
0.75	2	3.427	1.94	1.752–6.756
1.50	2	12.257	8.778	5.582–27.321
0.75	3	1.017	0.533	0.492–1.755
1.50	3	9.003	10.501	1.459–26.298
0.75	4	0.552	0.2	0.284–0.804
1.50	4	7.735	10.067	0.948–25.230
0.75	6	0.592	0.363	0.055–0.950
1.50	6	7.251	11.984	0.321–28.512
0.75	8	0.725	0.458	0.055–1.172
1.50	8	9.725	12.846	0.420–31.726

0.75	12	0.532	0.513	0.055–1.393
1.50	12	6.347	9.996	0.321–23.725
0.75	24	0.585	0.434	0.170–1.172
1.50	24	7.738	10.053	0.305–20.961
0.75	36	0.432	0.349	0.055–0.950
1.50	36	4.247	7.987	0.283–18.515
0.75	48	0.523	0.421	0.055–0.950
1.50	48	6.446	12.453	0.492–28.714
0.75	72	0.662	0.518	0.055–1.393
1.50	72	6.66	12.742	0.283–29.432
